# The Human Anger Face Likely Carries a Dual-Signaling Function

**DOI:** 10.3389/fnbeh.2018.00026

**Published:** 2018-02-20

**Authors:** Jinguang Zhang

**Affiliations:** Department of Communicology, University of Hawaii, Honolulu, HI, United States

**Keywords:** interpersonal aggression, anger, facial expression, physical strength, aggressive intent, costly signaling

## Introduction

Anger is an integral part of interpersonal aggression (Baumeister et al., 1990; Sell et al., 2009b) and has a cross-culturally recognizable facial expression (Ekman, 1973). This expression typically entails simultaneously lowering one's browridge, raising the cheeckbones and mouth, widening the nose, and pressing the lips (Ekman and Friesen, 1978; Sell et al., 2014). Given these species-typical features, recent studies sought to reveal their signaling function. That is, what does the human anger face communicate?

Sell et al. (2014) argued that the anger face mainly enhances facial cues of physical strength, thereby increasing the angry person's perceived fighting ability. In a paper published in the same year, Reed et al. (2014) argued that the anger face communicates the angry person's commitment to carry out threats. We believe that these two hypotheses complement each other to provide a more complete analysis of the signaling function of the anger face. In our discussion, we focus on men because interpersonal aggression is primarily a male activity (Puts, 2010). At the same time, though this opinion piece concerns the signaling function of face, we use research on vocal signals to build up our arguments. This is because (to our knowledge) a major component of this opinion piece, namely aggressive-intent signaling, has been mostly demonstrated with vocal signals.

### Aggressive signals

Aggressive signals are naturally-selected structures or acts that communicate signalers' threat potential, including their resource-holding potential (RHP; e.g., physical strength) and aggressive intent (i.e., the willingness to escalate in a fight; Hurd and Enquist, 2005). Both types of aggressive signals are prevalent in animals (see below), and the use of those signals helps reduce the cost of combats. For example, adult red deer stags weigh ~330 pounds on average and carry large, piercing antlers, and both features are capable of causing serious physical damages. However, the annual rate of permanent injuries is ~6% among stags that engage in rutting fights (Clutton-Brock et al., 1979). This is partly because roaring contests, where two stags stand apart from and take turn to roar at each other, resolve ~50% of the fights on average (Clutton-Brock and Albon, 1979). The roaring contests can resolve conflicts of interest because stags' roars convey information predictive of their chance of winning a pending fight against each other, and stags use such information to make fight-or-flight decisions.

#### Signals of RHP

Stags' roars are an RHP signal because the roaring rate correlates with stag's physical condition (e.g., deterioration caused by aging; Clutton-Brock and Albon, 1979) and the minimum formant frequency of the roars correlates with stags' body weight (Reby and McComb, 2003). With all else being equal, stags that are in better conditions and/or heavier are more likely to win physical fights. Importantly, only stags in better physical conditions can roar faster because roaring fast is energetically demanding. Stags in worse conditions may be able to roar faster than its condition allows, but this cannot last long and will quickly exhausts the stags, impairing their ability to make the next move. As such, an energetic cost proportional to signalers' condition prevents weaker stags from faking greater RHP, and the honesty of roaring rates as an RHP signal is maintained (i.e., the handicap principle; Zahavi, 1975). At the same time, because body weight is almost impossible to fake, stags' roars also constitute an “unfakeable” index signal of RHP (Maynard Smith and Harper, 2003).

#### Signals of aggressive intent

Aggressive-intent signals broadcast one's willingness to escalate in combats, and, as RHP signaling, animals that signal stronger aggressive intent are more likely to win the contested resources without fighting (Searcy and Nowicki, 2005). Much research showed that aggressive-intent signals exist (e.g., Vehrencamp, 2001; Searcy et al., 2006; Akçay et al., 2011), contrary to earlier arguments (e.g., Maynard Smith, 1982) that aggressive-intent signals could not have evolved. Those arguments are based on the observation that the association between the form of most aggressive-intent signals (e.g., song singing) and their content (e.g., aggressive intent) is often arbitrary. This would render the signals prone to bluff and thus useless in the long run in resolving conflicts of interest.

However, the retaliation-cost model (Enquist, 1985) suggests that aggressive-intent signals can be honest if they elicit aggression from signal receivers. Specifically, Enquist considered how an animal can use one of two signals, *S* (strong), and *W* (weak), to signal different levels of aggressive intent. *S* is more intense and more effective in repelling opponents than *W* is, and both strong and weak animals can use *S* and *W* equally well (i.e., the signals do not entail production or maintenance costs).

In the model, the focal strategy is: (1) if strong, signal *S*; if the opponent responds with *S*, attack; if the opponent signals *W*, repeat *S*, and attack if the opponent does not withdraw; but (2) if weak, signal *W* and give up if the opponent responds with *S*, but attack if the opponent signals *W*. Bluffing (i.e., using S when being weak) may succeed against weak opponents but will solicit attacks from strong opponents. When the cost to a weak animal of being attacked by a strong animal is larger than the benefit of bluffing, the focal strategy that promotes honest signaling (e.g., using *S* only when strong) can prevail against bluff and be selected.

Thus, the retaliation-cost model suggests that (1) aggressive-intent signals are honest when signal intensity is calibrated to signalers' RHP and (2) a receiver-dependent cost (i.e., retaliation) keeps deception rates low. Supporting the model, Anderson et al. (2012) showed that song birds higher in trait aggressiveness are more likely to approach opponents that emit soft songs, a putative aggressive-intent signal (see also, Popp, 1987; Molles and Vehrencamp, 2001). Recently, Zhang and Reid (2017) showed that men with greater threat potential (e.g., higher in upper-body strength) become more aggressive upon hearing a low-pitched male voice under a mating prime that simulates intrasexual competition. This finding suggests that the retaliation-cost model can be used to study human aggressive interactions.

### The case of the face

Facial muscles are highly homologous in anthropoids (Diogo et al., 2009). Like humans, several species of nonhuman primates (e.g., bonobos and chimpanzees) are capable of making facial expressions considered “angry” or threatening (Bard et al., 2011; Waller and Micheletta, 2013). For example, the bulging-lip face of chimpanzees is akin to the human anger face because they share muscle action units, including the chin raiser and lip pressor (Parr et al., 2007). The staring bared-teeth scream face, silent scream face, and the tense face of chimpanzees are threatening because those facial expressions are mostly observed in aggression initiators (Parr et al., 2005). However, whether those expressions signal chimpanzees' RHP, aggressive intent, or both remains unknown.

The human face is a reliable RHP signal, as men can accurately track other men's physical strength by looking at those men's neutral face (Sell et al., 2009a). Adding to the signaling function of the face, Sell et al. (2014) showed that the individual components of a prototypical anger face (e.g., lowered browridge) make the angry person appear physically stronger. The anger face also correlates with the angry person's approach tendencies (e.g., Yik, 1999; Adams et al., 2006). In particular, Reed et al. (2014) argued that the anger face signals the angry person's commitment to carry out threats by showing that proposers in an ultimatum game made more generous offers to a person making an angry face than to a person making a neutral face. Reed et al. further argued that the anger face honestly signals threat commitment because people only make the anger face when angry and that intense outburst of emotion makes its expression (i.e., the anger face) difficult to fake. Second, the complex neurological mechanisms associated with facial expressions also make an anger face difficult to fake. Collectively, this second line of research suggests that the anger face signals aggressive intent (Fridlund, 1994).

#### An integrative hypothesis

We believe that the cue-enhancement hypothesis (Sell et al., 2014) and the threat-commitment hypothesis (Reed et al., 2014) complement each other. Specifically, an anger face is scary (Fridlund, 1994), but behaviors only enhancing strength cues do not necessarily strike fear. For example, the movements that bodybuilders make in competitions should increase their perceived strength by emphasizing their upper-body muscles. However, audiences unlikely watch bodybuilding competitions with fear, because they know that the bodybuilders are not going to use that increased strength to attack them. In other words, the threat value (Searcy and Beecher, 2009) of aggressive signals will be reduced if intent cannot be reliably signaled and perceived.

We suspect that the anger face is intimidating because it signals the angry person's aggressive intent *in addition to* increasing the person's perceived strength by sending the message that “I am going to inflict this much physical force on you if you do not back off.” This message integrates the signaling functions specified by the cue-enhancement and threat-commitment hypotheses (i.e., fighting ability and intent). It also addresses a limitation of the threat-commitment hypothesis, that is, it is not clear from Reed et al. (2014) what the anger face commits in a threat. Given the link between the anger face and physical-strength perceptions (Sell et al., 2014), the commitment is likely about the amount of physical force one is ready to deploy in a pending fight.

If this is the case, a critical question is what maintains the honesty of the anger face as an aggressive signal. Reed et al. (2014) elaborated two mechanisms (see above), and we suggest two more here. The first is an energetic cost. Anger, at least when it is genuine, is tiring due to activities such as muscle contraction, blood vessel dilation, and increases in breathing and heart rates. These activities enhance one's physical strength, prepare one for a fight, but are also energetically demanding. It follows that only men in better physical conditions can more effectively mobilize their strength through angering, making their threat genuine and their anger face scary. Men in poorer physical conditions (e.g., being exhausted at the moment) can also assume an anger face, but the face is likely perceived as less intimidating. Compared to men in better conditions, those in poorer conditions should be less effective in mobilizing their physical strength, rendering their threat less genuine.

A retaliation cost can also help maintain the signal honesty of the anger face. To the extent the retaliation-cost model can be used to study human aggressive interactions (Zhang and Reid, 2017), the model predicts that an angrier face (compared to a less angry one) should be more likely to elicit aggression from physically stronger men than from physically weaker men. We are not aware of tests of this prediction. However, angry facial expressions were shown to induce approaching behaviors in observers in some studies (Adams et al., 2006; Wilkowski and Meier, 2010) but avoiding behaviors in others (Marsh et al., 2005; Seidel et al., 2010). The retaliation-cost model flags an unmeasured moderator: respondents' (i.e., signal receivers') physical strength. Approaching behaviors, which is associated with retaliation, should be more common among physically stronger respondents whereas avoidance should be more common among weaker respondents. Testing this moderation effect may help reconcile the seemingly inconsistent findings described above.

**Figure 1:**
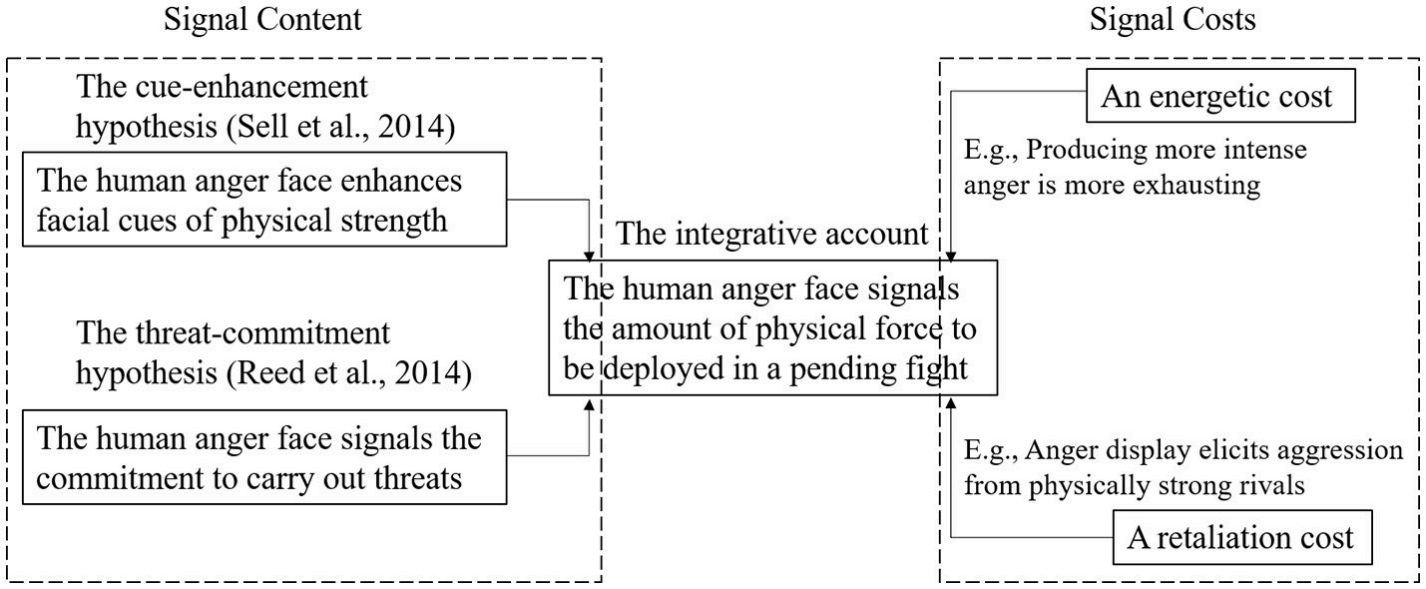
A schematic representation of the integrative account of the content and costs of the human anger face as an aggressive signal.

Figure 1 describes our integrative account of the signaling function of the human anger face in terms of its content and the costs that maintain its honesty.

This integrative hypothesis applies to other features of interpersonal aggression, too, such as violent yelling. To the extent that violent yells increase yellers' perceived physical strength (Sell, 2011), the yells may also carry a dual-signaling function like the anger face, that is, to advertise the amount of physical force one intends to inflict on opponents. At the same time, the honesty of violent yelling as an aggressive signal is also likely maintained by the energetic and retaliation costs. Testing these predictions will bridge human and animal models of anger and aggression and attest to the value of integrating those models in studying interpersonal aggression.

## Author contributions

JZ conceived, designed, and authored this research.

### Conflict of interest statement

The author declares that the research was conducted in the absence of any commercial or financial relationships that could be construed as a potential conflict of interest.
